# High-Speed Swimsuits and Their Historical Development in Competitive Swimming

**DOI:** 10.3389/fpsyg.2019.02639

**Published:** 2019-12-11

**Authors:** Alfonso Trinidad Morales, Javier Antonio Tamayo Fajardo, Higinio González-García

**Affiliations:** ^1^Faculty of Education and Humanities, Universidad Francisco de Vitoria (UFV), Madrid, Spain; ^2^Faculty of Education, Psychology and Sports Sciences, University of Huelva (UHU), Huelva, Spain; ^3^Faculty of Education, International University of la Rioja (UNIR), La Rioja, Spain

**Keywords:** fastskin, FINA, fabric, drag, records

## Abstract

The goal of this research was to review the experimental studies that have analyzed the influence of “high-speed swimsuits” on sports performance up to the appearance of the model “Jammer” in competitive swimmers. The design was a review following PRISMA Methodology, in which 43 studies were reviewed of a total of 512. Several searches were conducted in electronic databases of the existing research in this field (Google Scholar, Dialnet, Web of Sciences, and Scopus). The only studies excluded were those that reviewed the effects with neoprene and tests with triathletes. The studies that were included were published and peer-reviewed from 1999 to 2018 in which the effect of high-speed swimsuits was analyzed. The results showed the possible effects that high-speed swimwear can have in relation or not to competitive performance, biomechanical, physiological and psychological factors, flotation, drag, the material and the design until the introduction of the model “Jammer.” As conclusions, the lack of consensus due to the variety of fields of study means that improvements in competitions are still not clarified. In addition, the change in the rules may have effects on swimmers even though they have beaten records with other swimwear. Finally, the debate concerning whether medals were won unfairly or not is proposed.

## Introduction

Technological innovations have revolutionized the competitive swimwear industry (Mountjoy et al., 2009). Since the 1930s, baggy cotton swimsuits developed into smaller and tighter-fitting sizes, with the use of materials such as silk that absorbed less water than traditional woolen ones. Their aim was to reduce drag and improve performance, thus making swimmers faster (Foster et al., 2012). In the 80s, shorter models started to be used made of “Spandex” (Hagedorn, 2013), which were supposedly water-resistant. It was not until the end of the 90s that new cloth materials began to be developed, which newly offered less hydrodynamic drag than shaved skin. However, the development underwent a quantum leap with the introduction of polyurethane products, then cotton, silk, nylon and lycra (Davies, 1997). Even so, several studies were conducted in which the variables were the type of material, design and body coverage (Parsons and Day, 1986; Sharp and Costill, 1989; Toussaint et al., 1989; Cordain and Kopriva, 1991; Chatard et al., 1995; Starling et al., 1995; Trappe et al., 1995).

These appeared for the first time on an official basis at the Barcelona'92 Olympic Games, when the brand Speedo launched the model S2000 (Drašinac et al., 2015), known as the “Fast-Suit” (Craik, 2011). This was followed by the “Aquablade” (Atlanta '96), which was used by 77% of the winners of the said Olympiad (Parnell, 2008). Then, the Adidas full-body swimsuit in 1998 (Mountjoy et al., 2009) and the Speedo sharkskin “Fastskin” in 1999 (Drašinac et al., 2015), the latter proving to be a real revolution in Sydney 2000 (Farlex, 2000; Craik, 2005). Moreover, this swimsuit was the result of the joining of little grooves in “V” shape that reduced among 6 and 8% the total resistance. As a consequence, the “Ribblet effect” developed in 80's for Langley's Research Center from National Aeronautics and Space Administration (NASA), passed from apply in aeronaval industry (Takagi and Sanders, 2000) to swimming swimsuit after a decade. Known as first-generation swimsuits, they were designed to reduce drag and cover the entire body (Berthelot et al., 2010). However, they were not authorized by Fédération Internationale de Natation: International Swimming Federation (FINA) until 8 October 1999 (Craik, 2011; Foster et al., 2012) and in the run-up to the 2000 Olympic Games they revolutionized competitive swimming (Craik, 2011). Furthermore, in that year medals were won with and without these new swimsuits (Sanders et al., 2001).

In these games, the brand Speedo exaggerating the marketing claims of the “Fastskin,” guaranteed a reduction of 7.5% of the total drag along with an improvement of the swim time between 1 and 1.5 s every 100 m. However, there was a statistically insignificant reduction in drag (2%) with this type of swimsuit (*p* = 0.31) and not the one claimed by the manufacturer (Toussaint et al., 2002). However, there were doubts as to whether they would help swimmers to swim faster, mainly thanks to the fabric that supposedly repelled water in terms of “hydrophobicity.”

In 2004, the model Fastskin “FS-II” appeared, and subsequently the “FS-Pro” in 2007. But Speedo's biggest gamble did not arrive until February 2008 with the launch of the “LZR Racer” (Mountjoy et al., 2009), as it was said that it reduced drag thanks to the new polyurethane materials, to its strategically positioned panels on the body, the absence of seams and its texture based on shark scales. Moreover, it meant that in Beijing 2008 significant records were broken (Matheson, 2008; Craik, 2011) but it was denounced by “Arena” for “techno doping” (Parnell, 2008). Despite this, with time other brands (TYR, Nike, Mizuno, Asics, Blueseventy, Descente and Adidas) equaled that technology, producing a growing advertising war to capitalize on the potential benefits of gold medals and sponsorships (Craik, 2011).

Finally, in 2009 the swimsuits of the brands “Arena” and “Jaked” appeared, which were improved versions of the ones mentioned above, made 100% with polyurethane materials (Jaked J01 and Adidas Hydrofoil), which generated even more controversy and their use in competitions (Moloney, 2008a,b, 2009; Jeffery, 2009). However, as and from 1 January 2010 FINA changed the rules and authorized the model “Jammer” for men and the “Knee-length swimsuit” for women, similar to those used in the year 2000 (Foster et al., 2012).

Historically, between 2000, 2008, and 2009 were an improvement of performance in freely style around 1% in 50 m freestyle with the full body swimsuits (2000). After, performance increased between 1.5 and 3.5% with polyurethane paneled swimsuits (2008) and finally, 5.5% with full-body polyurethane ones (2009) (Foster et al., 2012). In addition, based on a review of the official times, an average improvement of ~2% was proven since the introduction of the new swimsuits (Mountjoy et al., 2009) for both sexes (Berthelot et al., 2010; Drašinac et al., 2015). Studies that analyzed competitions from 1990 to 2010, regarded that men (5.86%) and woman (5.57%) were faster in 100 m freestyle, according to the data issued by the IAAF (International Association of Athletics Federations) between 2010 and 2011 (O'Connor and Vozenilek, 2011). In a study on the generational evolution of results according to the type of swimsuit (Berthelot et al., 2010), the first generation showed a greater influence on women's events due to the compression of the upper part of the body, which was able to reduce the drag in that year, although this was not generalized in all of the events (Stager et al., 2001). However, men enhanced their performance in crawl between 0.9 and 1.4% with the use of full body swimsuit (Foster et al., 2012). The second generation produced great results with an average of 1.2 ± 0.5% (Men) and 1.0 ± 0.6% (Women) in all distances and styles, except in the 800 and 1,500 m freestyle. In the third generation, there was a major progression mainly among men and an improvement in the short distances for both genders (*p* < 0.05). As a whole, it was regarded that throughout generations females reached a similar increase, but with less performance comparing males in freestyle, which make more evident the advantage of swimsuits in sprint races (Foster et al., 2012). Conversely, in the long-distance events, together with the turns and the turbulences, it would produce a relative improvement depending on the distance swum (Men = 1.11 at 3.86% and Women = 0.87 at 3.74%), according to the averages obtained in the said study (Berthelot et al., 2010) and for the supposed rigidity of swimsuit that during competition caused tiredness to the swimmer (Foster et al., 2012).

After 10 world records were beaten in long-distance freestyle events with the swimsuits of the year 2000 and up to Beijing 2008, 108 new world records were set with the launch of the Speedo “LZR Racer,” of which 23 of the 25 new records were with the said model (Foster et al., 2012). In Rome 2009, 43 world records were set (Mountjoy et al., 2009), 15 in men's events and 17 in womens of the twenty events held in the 50-meter pool (Drašinac et al., 2015). But each of the men's records and 14 of the womens 17 records still stand (O'Connor and Vozenilek, 2011), according to the database of the magazine “SwimNews” (SwimNews, 2011). On the other hand, the controversies and their being dubbed as “technological doping” by the communications media, 25 and 47 world records were established in those years, respectively. Furthermore, and during the long-distance freestyle events, 17 records were set in 2008 and 16 more in 2009. Even so, nothing more was done after the shower of records as they continued to be used (Berthelot et al., 2010).

The brands have produced different models, adapting to the new trends, and have evolved according to the swimming style (Matsunami and Taimura, 2008), ensuring an improvement in the hydrodynamics and a reduction in drag (Foster et al., 2012). It is believed that new materials in combination with design have succeeded in improving buoyancy (Mountjoy et al., 2009; Wada et al., 2010). However, it is claimed that the textile has no effect on this (Chatard and Wilson, 2003; Roberts et al., 2003), unlike other studies (Förch et al., 2009; Mountjoy et al., 2009; Hagedorn, 2013). Materials such as “poliamide6” and “poliamide6-elastane,” have had a major effect on the results (Abasi et al., 2013) and the style (Issurin et al., 2014). The approximation of the fabric to so-called “shark skin and its scales,” has revealed an improvement in the circulation and a reduction in the turbulent fluid (Wada et al., 2010), together with the swimmer's drag (Oeffner and Lauder, 2012). Or even the superficial structure of the swimsuit (surface roughness, same and orientation) have revealed a significant effect on the aerodynamic/hydrodynamic drag (Moria et al., 2010, 2011c; Wada et al., 2010). However, the “Shark-Like” material of the FS-II2 model reduced the body rigidity drag by 7.7%, compared with the conventional swimsuit, under certain conditions (Benjanuvatra et al., 2002). Even, there were differences in favor of sharkskin swimsuits (−4.8 a 10.2%) in order to reduce the hydrodynamic resistance with different speeds (1.6 a 2.8 m/s), on surface as well as in 0.4 m deep compared to the traditional swimsuit in national swimmers. But in another study, the average drag coefficient (0.62 and 0.56) of swimsuits (FS-II and LZR) varied according to the speed (from 70 to 90 km/h) according to the experimental conditions applied (Moria et al., 2011a).

In this regard, it must be stressed that other analyses did not show a significant reduction in drag (Benjanuvatra et al., 2002; Toussaint et al., 2002), showing a minimal or nil effect when analyzing the textile weld of the model “LZR” (Moria et al., 2010). Even so, it is thought that polyurethane swimsuits produced an increase in swimming speed and constituted one of the most significant factors with 43 world records being set in a single championship (Berthelot et al., 2010; Neiva et al., 2011; O'Connor and Vozenilek, 2011; Cortesi et al., 2014). Also, what has lent solidity to these conclusions is the fact that full-body swimsuits reduce active (Benjanuvatra et al., 2002; Toussaint et al., 2002; Pendergast et al., 2006) and passive drag (Benjanuvatra et al., 2002; Pendergast et al., 2006; Smith et al., 2007). Conversely, no difference has been found compared with the conventional swimsuit, in relation to the position of the body segments to forward movement (Roberts et al., 2003; Wada et al., 2010), given that the design made the body shape more agile, reduced drag, compressed the muscles and controlled corporal deviations (O'Mahony and Braddock, 2002).

Although in the literature the effects of swimsuits have been analyzed from the shoulders to the ankles and the knees (e.g., Benjanuvatra et al., 2002; Chatard and Wilson, 2003; Roberts et al., 2003; Mollendorf et al., 2004), and from the waist to the ankles and the knees (Mollendorf et al., 2004). Body compression has been considered to be the main cause of the improvement in performance (Kainuma et al., 2009). Deformation could affect the swimming speed (Drašinac et al., 2015), taking into account that there are some which point out that the special fabric reinforcements in certain zones, together with the seamless fittings (LZR Racer, among others), could diminish the drag and push the body toward the surface, and in particular in the case of women (Berthelot et al., 2010), creating a hydrodynamic force (especially in the rear area or in the breasts). However, the average hydrostatic elevation would be smaller with polyurethane swimsuits, due to a reduction in the corporal volume but not in the mass. Furthermore, it could be influenced by the reduction in the chest/breast size and the abdominal circumference, suggesting that the improvement would not be related to static buoyancy and that it would even vary under dynamic conditions (Cortesi et al., 2010).

To all of this, one could add that excessive compression in the area of the thighs could alter the surface (Yermahanova et al., 2016), the area and the profile of the body (Sanders et al., 2001), reducing the size of the air pockets (Mountjoy et al., 2009), along with the muscular oscillations and skin vibration (Marinho et al., 2012), improving stroke coordination and frequency (Roberts et al., 2003; Chatard and Wilson, 2008). On the other hand, this compression could suppress blood circulation and the mitochondrial aerobic respiratory system (Kainuma et al., 2009), stimulating anaerobic glycolisis which would favor short distance events, due to the instantaneous strength of the white muscle fibers. On the other hand, the properties of polyurethane, as well as the tight fit and the fragility of the material have meant that putting it on is a real challenge, which requires an average of 30 and 45 min with the help of at least one or more assistants (Mountjoy et al., 2009). Other authors point out that some of them were impractical and even required between 15 and 20 min to put on, with the proviso that their durability would last for approximately half a dozen uses before they would lose their compression properties and effectiveness (Parnell, 2008; Craik, 2011).

Finally, another topic has been to find out whether the swimsuit has had any effect on drag and if it has really made a difference (Krieger, 2004; Moria et al., 2011a; Stefani, 2012). Based on that, Klauck and Llana (2003) did not find significant differences (*p* > 0.05) for the constant of drag in the passive resistance, nor in surface and neither in the underwater drag, as well as in the differentiation of gender in swimmers of national level. However, there has been no study that could prove a decline in dynamic resistance under controlled conditions (Oeffner and Lauder, 2012), although there was a fall of from 3 to 10% and from 10 to 15% under other study conditions (Mollendorf et al., 2004; Bixler et al., 2007). Nevertheless, one would have to take into account that swimming faster would mean increasing the thrust and reducing the drag, and that, furthermore, this seemed to be achieved with polyurethane swimsuits (Marinho et al., 2009). In addition, it is once again noted that coverage of the body (Benjanuvatra et al., 2002), together with the position of the body and the movements when confronted with water, could be some of the reasons why swimmers were faster (Kainuma et al., 2009) and this produced a reduction in drag (Neiva et al., 2011).

On the contrary, it is claimed that there is no evidence that full-body swimsuits reduce overall drag or friction, pressure and wave motion resistance (Toussaint et al., 2002; Pendergast et al., 2006; Wada et al., 2010; Van Geer et al., 2012). In any case, the role of friction resistance would be more prominent (Moria et al., 2010, 2011a; Foster et al., 2012), because it is more closely related to the properties of the swimmer's surface or the fabric, unlike the pressure and wave drag which are influenced by the body form. However, designs have sought to reduce active drag and increase buoyancy, in the hope of improving performance and the physiological cost at any speed (Roberts et al., 2003), although no physiological differences have been found (Smith et al., 2003).

Based on all of the foregoing, the aim of this research is to review all of the studies that have analyzed the influence of “high-speed swimsuits” up to the emergence of the “Jammer” among competitive swimmers, through the historical background, the materials, design, records, and the determining characteristics that place constraints on the swimmer.

## Materials and Methods

**Figure 1:**
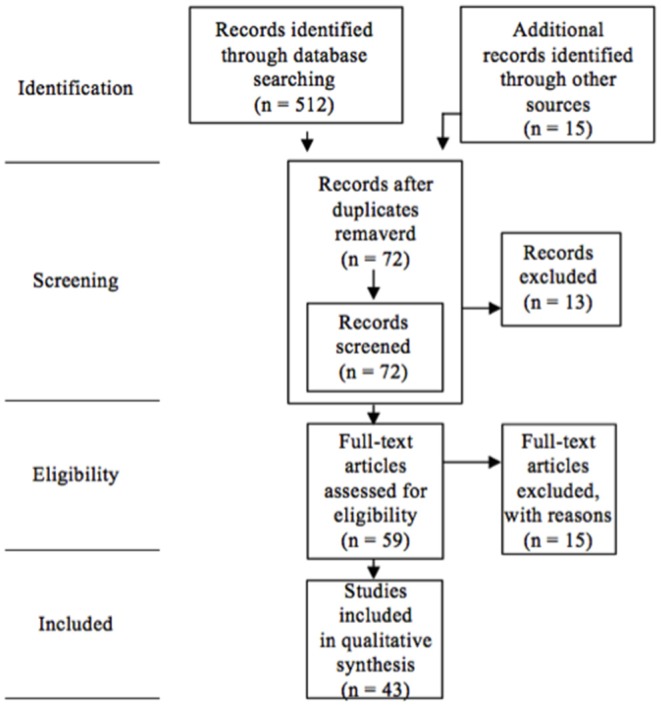
PRISMA flow chart (Moher et al., 2009; Carson et al., 2016).

A review was conducted following PRISMA Methodology (Moher et al., 2009; Carson et al., 2016), in which the main characteristics of the research papers that evaluate the effect of high-speed swimsuits on competitive swimming are described. To do this, an exhaustive search for research papers in this field was conducted. In order to prevent internal bias in individual studies and across studies were adopted the following criteria: that articles had been published from 8 October 1999, the date on which FINA authorized the use of the said swimsuits (Foster et al., 2012; Drašinac et al., 2015), until 2018, which was when the last search was conducted; the authors took into account the type of document, as only full-text articles written in English or another language (to reduce bias of revised publication) and peer-reviewed were taken into account (to ensure a minimum quality of the manuscript and a minimum reliability); that they analyzed the effect of swimsuits in historical, biomechanical, physiological and psychological terms; and they had been conducted using different types of swimsuit, related or not to performance. Furthermore, those that did not meet these criteria were excluded, as well as books, book chapters, theses, thesis reviews, presentations of monographical studies, notes of congresses, and studies that only reviewed the effects of neoprene suits (due to its use in open water swimming when the current work is focused on swimming indoor competitions) and triathletes events, but not the evolution of the high-speed swimsuit, its fabric and the different models, their effect on drag, buoyancy and the swimmer's characteristics, as well as their influence on different events, swimming events, and gender. Also, it is important to highlight that only were taken publications after searching exhaustively the existing researches of that field. Regarding that, four academic databases were used for the search for scientific material: Google Scholar, Dialnet, WOS (Web of Science) and Scopus (also with these data bases it is ensured a minimum quality to reduce bias). The search terms used were: “swimming swimsuit,” “high speed swimsuit,” “high tech swimsuit,” “bodysuit fastskin,” “fastskin swimming,” “fastskin suit,” “fastskin suit swimming,” “Speedo Arena suit,” “Speedo Arena bodysuit”; following boolean method to ensure that both terms were included in the articles. Searches were conducted by titles, abstracts and key words. In addition, lists of bibliographical references of the articles included in the said search were also reviewed. Also, review articles were not considered on the articles selection because they did not meet the rest of the criteria followed on this study. The first search that was carried out exhaustively produced a total of 512 records. Another article was identified by other means. Having deleted the duplicates, 72 documents were recorded, of which 13 were excluded, as they were not directly related to the aim of the study. Finally, 59 articles were reviewed, and once the inclusion criteria were applied, the 43 articles contained in this review were selected (Figure 1).

## Results

There have been many studies of swimsuits over a period of almost two decades (see Table 1). Not all of the analyses conducted within a research context could be extrapolated to high-level competitive environments. The possible effects on performance and their competitive development have also been measured.

**Table 1:** Main investigations that addressed the topic of fastskin swimsuits in chronological order.

**References**	**Title**	**Type of swimsuit**
Benjanuvatra et al. (2002)	Comparison of Buoyancy, Passive and Net Active Drag Forces Between Fastskin and standard swimsuits	Traditional Speedo Fastskin
Toussaint et al. (2002)	Effect of a fastskin body suit on drag during front crawl swimming	Fastskin Traditional
Roberts et al. (2003)	Effect of a fastskin suit on submaximal freestyle swimming	Speedo Fastskin Traditional
Mollendorf et al. (2004)	Effect of swimsuit design on passive drag	Speedo Fastskin (Shoulder/Ankle) Speedo Fastskin (Shoulder/Knee) Speedo Fastskin (Waist/Tobillo) Speedo Fastskin (Waist/Knee) Traditional Speedo
Smith et al. (2007)	The Influence of a compressive laminar flow body suit for use in competitive swimming	Traditional TYR Aquapel Laminar Flow Stitched
Chatard and Wilson (2008)	Effect of fastskin suits on performance, drag, and energy cost of swimming	Traditional Full Sleeveless Waist to Ankle
Kainuma et al. (2009)	Proposal of alternative mechanism responsible for the function of high-speed swimsuits	Speedo LZR Racer
Berthelot et al. (2010)	Technology and swimming: 3 steps beyond physiology	1st generation (1999) 2nd generation (2008) 3rd generation (2009)
Chollet et al. (2010)	Do Fastskin swimsuits influence coordination in front crawl swimming?	Arena Extrem Arena Powerskin-Revolution Speedo LZR Tyr Tracer Light Adidas JC Traditional
Cortesi et al. (2010)	The Effect of Wearing a Synthetic Rubber Suit on Hydrostatic Lift and Lung Volume	Traditional X-glide Power-skin Arena (Xg)
Keul et al. (2010)	Effects of new high-tech swimsuits on passive drag	Traditional Speedo LZR Racer Bluesenventy Nero Comp Arena R-Evolution
Moria et al. (2010)	Contribution of swimsuits to swimmer's performance	Speedo LZR Racer Speedo Fastskin-II (FS-II)
Silveira et al. (2010)	Effects of a Blueseventy Bodysuit on Spatial-Temporal and Coordinative parameters during an all-out 50 m front crawl stroke	Blueseventy Traditional
Wada et al. (2010)	An analysis of the underwater gliding motion in collegiate competitive swimmers	Traditional Speedo LZR Racer
Moria et al. (2011a)	Microstructures and aerodynamics of commercial swimsuits	Speedo LZR Speedo FSII Diana submarine Spalding full length TYR Sayonara
Moria et al. (2011b)	An evaluation of swimsuit performance	Diana Submarine (full body) Spalding full length
Neiva et al. (2011)	13th FINA World Championships: Analysis of swimsuits used by elite male swimmers	Jaked01 Full Powerskin X- Glide Full Powerskin X- Glide Pants LZR Racer Full Jaked01 Pants Hydrofoil Full LZR Racer Pants Unknown
Foster et al. (2012)	Influence of full body swimsuits on competitive performance	Full (2000) Speedo LZR (2008) Arena X-Glide (2009)
Marinho et al. (2012)	Effect of wearing a swimsuit on hydrodynamic drag of swimmer	Standard, Without Legs Latest Generation with Legs Light Underwear
Oeffner and Lauder (2012)	The Hydrodynamic Function of Shark Skin and Two Biomimetic Applications	Tissue Speedo Fastskin FS II Real Shark Skin
Van Geer et al. (2012)	Comparing swimsuits in 3D	Tres es de diferentes marcas y ajustables
Abasi et al. (2013)	Construction of drag force measuring system to characterize the hydrodynamics properties of swimsuit fabrics	Tissue of Nylon 6 Tissue of Nylon 6 Spandex Tissue of Polyester
Hagedorn (2013)	Physics of Swimming: Conditions that Affect the Passive Drag on a Swimmer in Streamline Position	Quick Jammer Regular Jammer
Chollet et al. (2014)	Evaluation of competitive jammers in expert male crawl swimmers	Jammers (A, B, C y D) of personal training
Cortesi et al. (2014)	Passive drag reduction using full-body swimsuits: the role of body position	Traditional X-glide Power-skin Arena R-Evolution + Powerskin Arena
Issurin et al. (2014)	Effect of high-tech swimsuits on the swimming performance in top-level swimmers	High Technology
Falcone et al. (2010)	Analysis of high-level swim performance in relationship with the introduction of new race swimsuits	Full Polyurethane

The main studies and conclusions in different areas based on the review are shown below: competitive performance, biomechanical, physiological and psychological effects, flotation, passive and active drag, materials and design (Tables 2, 3).

**Table 2:** Main investigations that have analyzed the effects of high-speed swimwear on competitive performance in chronological order.

**Authors**	**Competitive performance**
Stager et al. (2001)	- The swimsuits of the year 2000 did not have a generalized influence on swimming events
Smith et al. (2003)	- Swimsuits were not considered of any value by competitive male swimmers.
Roberts et al. (2003)	- There were no benefits in performance compared to traditional swimsuits in the case of unshaven swimmers
Matsunami and Taimura (2008)	- There were no benefits in the performance in swimming events compared with the conventional swimsuit
Smith et al. (2007)	- The swimsuit reduced drag (by 2%) at 2.0 m/s after the start (*p* = 0.002) and from the VO2 (*p*^∧^ 0.11), improving the time and the position of the swimmers in competition - The swimsuit reduced the swimming time (≈1 s) in the 50 m freestyle at maximum speed - The swimsuit reduced the swimming time (≈23 s) in the 1,500 m freestyle at competition speed
Chatard and Wilson (2003)	- There were substantial differences in performance with the swimsuit compared with the conventional type - The swimsuit produced in an increase in stroke length and cut swimming time in the crawl
Kainuma et al. (2009)	- Compression favored short-distance events due to the instantaneous force of the white muscle fibers
Berthelot et al. (2010)	- The change in the regulations led to a return to the performance thresholds of 1999 to 2007 - In 2000, 2008, and 2009, performance improved by 2.0% for both genders
Neiva et al. (2011)	- There are preferences for full-body swimsuits (Powerskin X Glide Full and Jaked01 Full) - Use varied according to the model: Jaked01 Full (47.07%), Powerskin X-Glide (34.56%), Powerskin X Glide Pants (7.35%) and LZR Racer Full (5.15%) - In crawl events most men wore full-body swimsuits - El Jaked01 Full was used by 62.5 and 25% of male and female swimmers, respectively, while the Powerskin X-Glide Full was used by 37.5 of male swimmers and 62.5% of female swimmers in the 50 and 100 m
Foster et al. (2012)	The swimsuits of the year 2000 improved performance among men in the crawl by between 0.9 and 1.4% - The 2008 swimsuits increased performance by between 1.5 and 3.5% - The 2009 swimsuits increased performance by up to 5.5% - The advantage in performance was more evident in the speed events
Issurin et al. (2014)	- Swimsuits were an aid to performance in 50 m events in both genders - Swimsuits are an aid to performance in low-drag events (crawl and backstroke) in contrast to those with high-drag (breastroke and butterfly) - Rejection of high-tech swimsuits reduced sports performance
Falex	- The swimsuit had an influence on improved performance in all swimming specialities
**Authors**	**Flotation**
Benjanuvatra et al. (2002)	- The Fastskin swimsuit did not increase buoyancy
Roberts et al. (2003)	- The improvement in swimsuit performance is not related to static buoyancy
Chatard and Wilson (2003)	- There were no buoyancy effects on the five brands that used sharkskin material
Mountjoy et al. (2009)	- The swimsuit's new materials and the design improved its buoyancy in water
Cortesi et al. (2014)	- The improvement in swimsuit performance is not related to static buoyancy
Wada et al. (2010)	- The LZR swimsuit with a thrust of up to 1.82 m at 0.8 s slightly altered buoyancy

**Table 3:** Main research studies that have analyzed the effects of high-speed swimsuits on biomechanical, physiological, psychological effects, drag, materials, and designs, in chronological order.

**Authors**	**Biomechanical, physiological and psychological effects**
Roberts et al. (2003)	- There were no physical, psychological or biomechanical benefits with the swimsuit in the crawl at top speed compared with the conventional suit - Psychological effects on performance are not ruled out and would explain a greater stroke length
Smith et al. (2007)	- There were no differences among the swimsuits in terms of oxygen consumption in distances of over 400 m
Matsunami and Taimura (2008)	- There were no physiological differences from the traditional women's swimsuit and the neck to ankle type
Chatard and Wilson (2003)	- The energy cost, the perception of effort and concentrations of lactic acid were less with the full-body suit and waist-high suit than with the conventional suit during the sub-aquatic phase
Kainuma et al. (2009)	- The swimsuit stimulates the anaerobic glucolysis system - The swimsuit suppresses blood circulation and the mitochondrial aerobic respiratory system
Wada et al. (2010)	- The LZR after thrusting up to 1.82 m and at 0.8 s slightly altered slippage, angular alignment (knee/hip at 180°) and the generation of turbulences
Foster et al. (2012)	- Fatigue due to rigid swimsuits reduces performance in long distance events due to the turns
Van Geer et al. (2012)	- Distortion of the body due to the swimsuit influences performance and reduces drag
**Authors**	**Passive and active drag**
Benjanuvatra et al. (2002)	- The swimsuit appeared to reduce overall hydrodynamic drag compared with the traditional one.
Toussaint et al. (2002)	- There was no significant reduction in drag (2%) with the Fastskin (*p* = 0.31) - The 7.5% reduction in drag claimed by the manufacturer was not corroborated
Smith et al. (2003)	- The undertow force did not diminish in the sharkskin suit compared with the conventional suit
Mollendorf et al. (2004)	- Swimsuits from the shoulders to the knees and the ankles slightly reduced passive drag - The increase in friction against the upper part of the body reduced the pressure and wave drag from the shoulders to the knees - Swimsuits from the shoulders reduced drag and improved performance at 1.5 m/s - Swimsuits from the torso and the legs reduced drag and improved performance
Chatard and Wilson (2003)	- Full-body and waist to ankle swimsuits reduced passive drag in comparison with conventional suits
Moria et al. (2011a)	- A high-speed swimsuit reduces drag by approximately 35%
Hagedorn (2013)	- Energy is lost due to drag (48%) with a conventional swimsuit compared to a high-speed suit
Cortesi et al. (2014)	- A full-body swimsuit reduces passive drag due to the composition and changes of position
**Authors**	**Materials and design**
Smith et al. (2007)	- The adjustable and compressive laminar flow swimsuit may improve performance
Moria et al. (2011c)	- The drag coefficient varied in the FSII (≈0.62 a <70 km/h) and LZR (≈0.56 a <90 km/h) - The fiber orientation and textile weld have a minimal or no effect on the hydrodynamics
Wada et al. (2010)	- The LZR after a thrust of up to 1.82 m and 0.8 s slightly altered friction as it was seamless
Moria et al. (2011a)	- A notable variation exists in the hydrodynamic properties among swimsuits - Swimsuit fabric improves the flow transition at low speeds - Roughness, seaming and orientation have an effect on aero/hydrodynamic drag
Oeffner and Lauder (2012)	- There was no increase in swimming speed with Speedo fabric - Swimming speed increased with the riblets (7.2%) and sharkskin (12.3%) - Shark skin denticles could improve swimming speed and reduce drag
Van Geer et al. (2012)	- The surface of the swimsuit also contributes to the reduction in resistance
Abasi et al. (2013)	- The fabric surface and its design play a prominent role in the swimmer's performance
Hagedorn (2013)	- The textile hydrophobicity of the fast Jammer is greater because it has a surface that is much less resistant to water and this will allow faster and longer transfer lines - The age of the swimsuit plays an important role in the textile's hydrophobicity to water

## Discussion

The aim of the study was to review the research studies that have analyzed the use of high-speed swimsuits and their historical development in competitive swimming up to the Jammer model. Historical aspects, analysis of competitive results, materials and designs, as well as the determining characteristics of the swimmer and their effect on swimming, were the topics on which the study focused.

The historical background revealed a great concern with reducing drag by using the most appropriate swimsuit for the period (Foster et al., 2012; Hagedorn, 2013) and for the material (Matsunami and Taimura, 2008; Abasi et al., 2013; Issurin et al., 2014), to bring about a significant improvement thanks to technological innovations (Mountjoy et al., 2009). Since the 1930s a variety of studies had been carried out, mostly with triathletes (Parsons and Day, 1986; Sharp and Costill, 1989; Toussaint et al., 1989; Cordain and Kopriva, 1991; Chatard et al., 1995; Starling et al., 1995; Trappe et al., 1995), in which the conclusions were a long way from extrapolated to the competitive swimming of the time, before FINA approved the use of high-speed swimsuits in 1999, also known as technological or modified swimsuits (Craik, 2011; Foster et al., 2012).

From Barcelona ′92 to Rome 2009, there was a generational improvement (Parnell, 2008; Mountjoy et al., 2009; Berthelot et al., 2010; Craik, 2011; Foster et al., 2012; Drašinac et al., 2015) that revolutionized competitive swimming over almost two decades, causing some swimmers to consider whether to use them or not, regardless of the results and the medals obtained (Sanders et al., 2001), even when faced with a generalized contrary effect (Stager et al., 2001). Even so, the improvement in terms of times and records that still stand (O'Connor and Vozenilek, 2011; SwimNews, 2011) has been demonstrated in a significant and longitudinal manner, despite the controversies received (Moloney, 2008a,b, 2009; Jeffery, 2009). However, nobody has associated any of these improvements in times with other advances in terms of research, training, physiology or even ergogenic aids, among others. Nevertheless, the change in the regulations has led to a reconsideration as to whether current performance is closer to that of the year 2000 or if there is even are turn to the thresholds that applied between 1999 and 2007 (Berthelot et al., 2010). Therefore, the question arises as to whether they helped during the race or solely to inflate records and raise the bar.

Brands have also produced different models (Matsunami and Taimura, 2008) and used different materials that have improved hydrodynamics (Moria et al., 2010; Wada et al., 2010; Foster et al., 2012), flotation (Förch et al., 2009; Mountjoy et al., 2009; Wada et al., 2010; Hagedorn, 2013), competitive results (Berthelot et al., 2010; Neiva et al., 2011; O'Connor and Vozenilek, 2011; Abasi et al., 2013; Cortesi et al., 2014) and have reduced active and passive drag (Pendergast et al., 2006; Smith et al., 2007), even adapting models to swimming style (Issurin et al., 2014). However, for the scientific community, materials have not played a determining role in reducing drag (Benjanuvatra et al., 2002; Toussaint et al., 2002; Chatard and Wilson, 2003; Roberts et al., 2003; Moria et al., 2011b). The vast majority of studies have not taken swimsuit design into account when extrapolating their results (Benjanuvatra et al., 2002; Mollendorf et al., 2004; Moria et al., 2010, 2011c; Abasi et al., 2013; Issurin et al., 2014), as to date it has not been possible to prove a reduction in drag in dynamic and controlled conditions, where the swimming speed and the hydrodynamics of the fabric could be compared quantitatively a superficial level (Oeffner and Lauder, 2012). It must be taken into account that design, supposedly, produced certain advantages, and based on this, they were progressively adapted (Chatard and Wilson, 2003; Berthelot et al., 2010; Neiva et al., 2011; Foster et al., 2012) revealing a better effect on the crawl and backstroke, as they offered less drag to the breaststroke and butterfly (Issurin et al., 2014). However, the distance of the race, turns and turbulences can also influence swimming time (Chatard and Wilson, 2003), as swimsuits may have restricted movement in long races, but not in short, fast races (Berthelot et al., 2010; Foster et al., 2012). Consequently, the preferences of the sprint swimmer, middle-distance and long-distance swimmers, together with the style led to different models and brands being used, which converted the start control area into a veritable circus of colors and shapes, although today only the formal aspect has been regulated. Furthermore, the issues of these models are that they take quite a long time to put on (15 to 45 min), and that they are fragile, with only transitory durability (Parnell, 2008; Mountjoy et al., 2009; Craik, 2011).

It should be added that among so much controversy about textiles there were several disputes. The demands of the brands on the designs and the material were not the same for all manufacturers, thus revealing differences between their resources (Parnell, 2008; Mountjoy et al., 2009; Craik, 2011; Drašinac et al., 2015). This entailed an unfair advantage that was determined by the price and the swimmer's resources. Another observation is that brands attempted to demonstrate the benefits of their products in theoretical terms in order to lead sales, advertising and sponsorship, going so far as to exaggerate their properties (Sanders et al., 2001; Craik, 2011). This resulted in the manufacturers being accused of techno doping (Parnell, 2008), which is today one of the main stains that tarnish so many sports, including swimming.

Equally, the effect on body coverage has posed the questions of how this could affect the swimmer and his or her swimming style (Benjanuvatra et al., 2002; Roberts et al., 2003; Mollendorf et al., 2004; Förch et al., 2009). There has been more discussion of a distortion (Drašinac et al., 2015) than compression of the body, which has opened up a far-reaching analysis (Sanders et al., 2001; Chatard and Wilson, 2003; Roberts et al., 2003; Kainuma et al., 2009; Marinho et al., 2009; Mountjoy et al., 2009; Cortesi et al., 2010; Yermahanova et al., 2016). The conclusions drawn confirm that both performance and speed could be affected (Kainuma et al., 2009; Drašinac et al., 2015). Moreover, they point out that women could benefit compared with men thanks to the compression and body composition (Berthelot et al., 2010; Cortesi et al., 2010), despite the fact that generally the opposite is supposedly the case (Berthelot et al., 2010; O'Connor and Vozenilek, 2011). However, there have been few studies on the differentiation between genders that could corroborate these data (Drašinac et al., 2015). Also, the said compression could affect drag and reduce it (Foster et al., 2012) thanks to an improvement in hydrodynamics and buoyancy (Cortesi et al., 2010), given that the extent to which the swimsuit adjusts to the body, it at all, could influence the swimming and the performance in the race compared with a swimsuit of a different kind (Benjanuvatra et al., 2002; Roberts et al., 2003; Mollendorf et al., 2004; Wada et al., 2010). Again, and due to the shortage of data, more studies are needed that would corroborate whether the anthropometric characteristics and the skills of the swimmer could be decisive, since in competitions not all obtain the same results (Sanders et al., 2001). Furthermore, the question arises as to whether the repeal of the regulation has not had the same impact and could affect performance and swimming as a sporting event.

Another highly controversial topic was the finding of the reduction in drag and as a result the improvement in performance and results (Benjanuvatra et al., 2002; Krieger, 2004; Mollendorf et al., 2004; Pendergast et al., 2006; Bixler et al., 2007; Marinho et al., 2009; Moria et al., 2010, 2011c; Foster et al., 2012; Stefani, 2012; Van Geer et al., 2012). The differences of opinion and conclusions triggered heated debate among scientists, and not only because of ulcers on the fingertips and distal interphalangeal joints, in addition to ecchymosis on the lower limbs (Mountjoy et al., 2009), which were allegedly caused by the use of the swimsuit, but also due to the wide range of studies and analysis conditions that did not lead to a consensus, raising doubts about the possible biomechanical, physiological and psychological improvements (Chatard and Wilson, 2003; Roberts et al., 2003; Smith et al., 2003; Matsunami and Taimura, 2008; Kainuma et al., 2009; Wada et al., 2010; Foster et al., 2012), as well as in competition (Farlex, 2000; Stager et al., 2001; Smith et al., 2007; Berthelot et al., 2010; Neiva et al., 2011; Issurin et al., 2014), with few studies on dynamic drag (Oeffner and Lauder, 2012). Even so, these swimsuits continue to be used in training to reach supramaximal speeds and to understand their influence on drag (Cortesi et al., 2014).

Finally, we must not forget that the FINA prohibits any device that could help speed, buoyancy and/or resistance during a competition. It generated a major debate until the other types of swimsuits were approved (Moloney, 2008a,b, 2009; Jeffery, 2009) and the pre-2010 models were withdrawn, which made the influence on performance evident (O'Connor and Vozenilek, 2011). Furthermore, one may well ask why they continued to be used during those two decades and why there was not a massive withdrawal. This may be related to a contradiction in the regulations or it may be related to the brands' sponsors, as it is well-known that they are present in all of the advertising and publicity that encompass the competitions in general. Hence, there may have been a deliberate use during these three generations, even though today with the new models most of those records have been beaten, disproving the supposed benefits and improvements that they were claimed to produce. Today's models do not have the same characteristics as their predecessors, and this may even have been a factor in their impact on competition.

As future lines of research, we would propose conducting studies on the current models that would analyse their possible influence on dynamic drag, due to the current lack of data. It would also be useful to conduct a comparative study to find out whether the times set since the use of first-generation swimsuits (at the Sydney 2000 Olympics) have any relation to the “Jammer” model, in view of the drop in performance that some researchers have claimed to observe.

Regarding the limitations of this study, it was solely focused on high-speed swimsuits since their approval in 1999, and therefore it could have been useful to know the transferences from other subsequent studies of first-generation swimsuits.

## Conclusions

Controversies exist as there are those who believe fervently that these swimsuits have had some benefit for performance in general, according to the gender, the distance, the style and the physical properties of the swimmer, thanks to the models, materials and fabrics used by the manufacturers. It is said that some benefited from a placebo effect on performance and that the change in the regulations may have had other psychological effects on such high-level swimmers, although they have beaten world records, improved times and raised the bar in terms of times. But beyond all of this, one is faced with the moral dilemma of knowing that medals were won, and world records were beaten, being above the limits of human performance. However, the lack of evidence to clarify such opinions places any consensus among researchers in doubt, despite the years that have passed.

## Practical Implications

The key aspects that emerge from the study we have conducted are set out below:

- The importance of materials in sporting performance.
- The need for a body within the federations (or an external body) that “oversees” and monitors advances in the use of materials, as any such advances could have a decisive influence on performance, in order to prevent “technology doping” and respond to new developments as they arise in this sphere.
- The need for scientific studies that can look into what exactly it is that brands want to “sell” without research results to back it up.
- Investigate whether sports sponsorship may not be an obstacle to put a stop to “technology doping.”

## Author Contributions

AM conducted the methodology, introduction, and writing. JT did part of the introduction. HG-G provided methodological support and checked the writing style and references.

### Conflict of Interest

The authors declare that the research was conducted in the absence of any commercial or financial relationships that could be construed as a potential conflict of interest.

## References

[B1] Abasi S. Nasrollahi T. Aghajani M. Tehran M. A. (2013). Construction of drag force measuring system to characterize the hydrodynamics properties of swimsuit fabrics. J. Indust. Textiles 43, 264–280. 10.1177/1528083712452901

[B2] Benjanuvatra N. Dawson G. Blanksby B. A. Elliott B. C. (2002). Comparison of buoyancy, passive and net active drag forces between fastskin and standard swimsuits. J. Sci. Med. Sport 5, 115–123. 10.1016/S1440-2440(02)80032-9 12188083

[B3] Berthelot G. Len S. Hellard P. Tafflet M. El Helou N. Escolano S. (2010). Technology and swimming: 3 steps beyond physiology. Mater. Today 13, 46–51. 10.1016/S1369-7021(10)70203-0

[B4] Bixler B. Pease D. Fairhurst F. (2007). The accuracy of computational fluid dynamics analysis of the passive drag of a male swimmer. Sports Biomech. 6, 81–98. 10.1080/14763140601058581 17542180

[B5] Carson V. Hunter S. Kuzik N. Wiebe S. A. Spence J. C. Friedman A.. (2016). Systematic review of physical activity and cognitive development in early childhood. J. Sci. Med. Sport. 19, 573–578. 10.1016/j.jsams.2015.07.011 26197943

[B6] Chatard J. C. Senegas X. Selles M. Dreanot P. Geyssant A. (1995). Wet suit effect: a comparison between competitive swimmers and triathletes. Med. Sci. Sports Exerc. 27, 580–586. 10.1249/00005768-199504000-00017 7791590

[B7] Chatard J. C. Wilson B. (2003). Drafting distance in swimming. Med. Sci. Sports Exerc. 35, 1176–1181. 10.1249/01.MSS.0000074564.06106.1F 12840639

[B8] Chatard J. C. Wilson B. (2008). Effect of fastskin suits on performance, drag and energy cost of swimming. Med. Sci. Sports Exerc. 40, 1149–1154. 10.1249/MSS.0b013e318169387b 18460989

[B9] Chollet D. Chavallard F. Lemaitre F. Seifert L. (2010). Do Fastskin swimsuits influence coordination in front crawl swimming?, Paper Presented at the XIth International Symposium for Biomechanics and Medicine in Swimming (Oslo).

[B10] Chollet D. Puel F. Marinho D. Ramos R. Lepretre P. Louvet B. (2014). Evaluation of competitive jammers in expert male crawl swimmers, in XIIth International Symposium for Biomechanics and Medicine in Swimming (Canberra), 95–100.

[B11] Cordain L. Kopriva R. (1991). Wetsuits, body density and swimming performance. Br. J. Sports Med. 25, 31–33. 10.1136/bjsm.25.1.31 1913028 PMC1478803

[B12] Cortesi M. Fantozzi S. Di Michele R. Zamparo P. Gatta G. (2014). Passive drag reduction using full-body swimsuits: The role of body position. J. Strength. Cond. Res. 28, 3164–3171. 10.1519/JSC.0000000000000508 24796982

[B13] Cortesi M. Zamparo P. Tam E. da Boit M. Gatta G. (2010). The effect of wearing a synthetic rubber suit on hydrostatic lift and lung volume. Biomechan. Med. Swim. XI, 57–59.

[B14] Craik J. (2005). Fashioning Australian bodies and national culture, in Dressed, Dressed up, Undressed. On Clothes, Body and Identity, 1st Edn, ed Nilsson B. G. (Stockholm: Norstedts Akademiska Forlag, 73–110.

[B15] Craik J. (2011). The fastskin revolution from human fish to swimming androids. Cult. Unbound J. Curr. Cult. Res. 3, 71–82. 10.3384/cu.2000.1525.11371

[B16] Davies E. (1997). Engineering swimwear. J. Textile Inst. 88, 32–36. 10.1080/00405009708658585

[B17] Drašinac G. Karninčić H. Jašić D. Burger A. (2015). Environmental success factors or the justification for the prohibition of high-tech swimsuits in swimming. Coll. Antropol. 39, 181–184. 26434028

[B18] Falcone L. Nagni G. Demarie S. (2010). Analysis of high-level swim performance in relationship with the introduction of new race swimsuits. Sport Sci Rev. XIX, 177–186. 10.2478/v10237-011-0011-1

[B19] Farlex (2000). SPEEDO Introduces Fastskin-The Fastest Swimsuit Ever Made. Business Wire. Retrieved from: http://www.thefreelibrary.com/SPEEDO+Introduces+Fastskin+---+the+Fastest+Swimsuit+Ever+Made.-a060827273. (accessed September 28, 2017).

[B20] Förch R. Schönherr H. Jenkins A. T. A. (2009). Surface Design: Applications in Bioscience and Nanotechnology. Weinheim: Wiley VHC verlag GmbH and Co. KGaA.

[B21] Foster L. James D. Haake S. (2012). Influence of full body swimsuits on competitive performance. Proc. Eng. 34, 712–717. 10.1016/j.proeng.2012.04.121

[B22] Hagedorn E. (2013). Physics of Swimming: Conditions that Affect the Passive Drag on a Swimmer in Streamline Position. Wooster, OH: Physics Department, The College of Wooster.

[B23] Issurin V. Issurin V. Pushkar-Verbitsky V. Pushkar-Verbitsky V. Verbitsky O. Verbitsky O. (2014). Effect of high-tech swimsuits on the swimming performance in top-level swimmers. J. Sports. Med. Phys. Fitness 54, 383–388. 24598554

[B24] Jeffery N. (2009). Rice well-placed in swimsuit wars. The Weekend Australian. 2–3.

[B25] Kainuma E. Watanabe M. Tomiyama-Miyaji C. Inoue M. Kuwano Y. Ren H.. (2009). Proposal of alternative mechanism responsible for the function of high-speed swimsuits. Biomed. Res. 30, 69–70. 10.2220/biomedres.30.69 19265266

[B26] Keul S. Bieder A. Wahl P. (2010). Effects of new high-tech swimsuits on passive drag, A paper presented at the XIth International Symposium for Biomechanics and Medicine in Swimming (Oslo), 16–19.

[B27] Klauck J. Llana S. (2003). Bañadores tradicionales vs bañadores de última generación: Efecto sobre la resistencia pasiva. Comun. Técn. 4, 53–55.

[B28] Krieger K. (2004). Do pool sharks swim faster? Science 305, 636–637. 10.1126/science.305.5684.636 15286360

[B29] Marinho D. A. Barbosa T. M. Kjendlie P. L. Vilas Boas J. P. Alves F. B. Rouboa A. I. (2009). Swimming simulation: a new tool for swimming research and practical applications, in Computational Fluid Dynamics for Sport Simulation. Lecture Notes in Computational Science and Engineering, ed Peters M. (Heidelberg; Berlin: Springer), 33–61.

[B30] Marinho D. A. Mantha V. R. Vilas-Boas J. P. Ramos R. J. Machado L. Silva A. J. (2012). Effect of wearing a swimsuit on hydrodynamic drag of swimmer. Braz. Arch. Biol. Technol. 55, 851–856. 10.1590/S1516-89132012000600007

[B31] Matheson C. (2008). “Speedo Makes Waves at Olympics”. Available online at: http://news.bbc.co.uk/2/hi/business/7558622.stm (accessed November 8, 2018).

[B32] Matsunami M. Taimura A. (2008). Trend to swimsuit choices of male swimmers in the competition from 2001 to 2007: 2186. Med. Sci. Sports Exerc. 40:S398 10.1249/01.mss.0000322698.16421.93

[B33] Moher D. Liberati A. Tetzlaff J. Altman D. G. The P. R. I. S. M. A. Group (2009). Preferred reporting items for systematic reviews and meta-analyses: the PRISMA statement. PLoS Med. 6:e1000097 10.1371/journal.pmed.1000097 19621072 PMC2707599

[B34] Mollendorf J. C. Termin I. I. A. C. Oppenheim E. Pendergast D. R. (2004). Effect of swim suit design on passive drag. Med. Sci. Sports Exerc. 36, 1029–1035. 10.1249/01.MSS.0000128179.02306.57 15179173

[B35] Moloney J. P. (2008a). Call for ruling on super suits, in The Canberra Times (Canberra), 29.

[B36] Moloney J. P. (2008b). Officials strip juniors of state-of-the-art suits, in The Canberra Times (Canberra), 20.

[B37] Moloney J. P. (2009). FINA's credibility crisis could have been avoided, if it had remembered…the suit that never was… in The Canberra Times (Canberra).

[B38] Moria H. Chowdhury H. Alam F. (2011a). Microstructures and aerodynamics of commercial swimsuits. Proc. Eng. 13, 389–394. 10.1016/j.proeng.2011.05.103

[B39] Moria H. Chowdhury H. Alam F. (2011b). An evaluation of swimsuit performance. Proc. Eng. 13, 382–388. 10.1016/j.proeng.2011.05.102

[B40] Moria H. Chowdhury H. Alam F. Subic A. (2011c). Aero/hydrodynamic study of speedo LZR, TYR sayonara and blueseventy Pointzero3 swimsuits. Jordan J. Mech. Ind. Eng. 5, 83–88.

[B41] Moria H. Chowdhury H. Alam F. Subic A. Smits A. J. Jassim R. (2010). Contribution of swimsuits to swimmer's performance. Proc. Eng. 2, 2505–2510. 10.1016/j.proeng.2010.04.023

[B42] Mountjoy M. Gordon I. McKeown J. Constantini N. (2009). Medical complications of an aquatic innovation. Br. J. Sports Med. 43, 979–980. 10.1136/bjsm.2009.067215 19858109

[B43] Neiva H. P. Vilas-Boas J. P. Barbosa T. M. Silva A. J. Marinho D. A. (2011). 13th fina world championships: Analysis of swimsuits used by elite male swimmers. J. Hum. Sport Exerc. 6, 87–93. 10.4100/jhse.2011.61.10

[B44] O'Connor L. M. Vozenilek J. A. (2011). Is it the athlete or the equipment? an analysis of the top swim performances from 1990 to 2010. J. Strength Cond. Res. 25, 3239–3241. 10.1519/JSC.0b013e3182392c5f 21964430

[B45] Oeffner J. Lauder G. V. (2012). The hydrodynamic function of shark skin and two biomimetic applications. J. Exp. Biol. 215, 785–795. 10.1242/jeb.063040 22323201

[B46] O'Mahony M. Braddock S. E. (2002). Sportstech: Revolutionary Fabrics, Fashion and Design. London: Thames and Hudson.

[B47] Parnell S. (2008). Slippery business. The Australian, 31. Available online at: http://www.theaustralian.com.au/news/features/slippery-business/story-e6frg8h6-1111116490096 (accessed May 31, 2018).

[B48] Parsons L. Day S. J. (1986). Do wet suits affect swimming speed? Br. J. Sports Med. 20, 129–131. 10.1136/bjsm.20.3.129 3779341 PMC1478371

[B49] Pendergast D. R. Mollendorf J. C. Cuviello R. Termin A. C. (2006). Application of theoretical principles to swimsuit drag reduction. Sports Eng. 9, 65–76. 10.1007/BF02844859

[B50] Roberts B. S. Kamel K. S. Hedrick C. E. McLean S. P. Sharp R. L. (2003). Effect of a FastSkin suit on submaximal freestyle swimming. Med. Sci. Sports Exerc. 35. 519–524. 10.1249/01.MSS.0000053699.91683.CD 12618585

[B51] Sanders R. B. Rushall H. Toussaint J. Stager J. (2001). Bodysuit yourself, but first think about it. J. Turbul. 3

[B52] Sharp R. L. Costill D. L. (1989). Influence of body hair removal on physiological responses during breaststroke swimming. Med. Sci. Sports Exerc. 21, 576–580. 10.1249/00005768-198910000-00013 2691818

[B53] Silveira R. P. Kanefuku J. Y. More F. C. Castro F. A. S. (2010). Effects of a blueseventy bodysuit on spatial-temporal and coordinative parameters during an all-out 50-M front crawl stroke, in XIth International Symposium for Biomechanics and Medicine in Swimming (Oslo: Norwegian School of Sport Sciences), 102–103.

[B54] Smith J. W. Molloy J. M. Pascoe D. D. (2003). the efficacy of body suits at reducing drag and oxygen cost in competitive swimming. Med. Sci. Sports Exerc. 35(Suppl. 1):S97 10.1097/00005768-200305001-00537

[B55] Smith J. W. Molloy J. M. Pascoe D. D. (2007). The influence of a compressive laminar flow body suit for use in competitive swimming. J. Swim. Res. 17, 10–16.

[B56] Stager J. M. Skube J. Tanner D. A. Winston W. Morris H. H. (2001). Predicting elite swim performance at the USA 2000 Olympic swim trails. Med. Sci. Sports Exerc. 33:S159 10.1097/00005768-200105001-00898

[B57] Starling R. D. Costill D. L. Trappe T. A. Jozsi A. C. Alison C. Trappe S. W.. (1995). Effect of swimming suit design on the energy demands of swimming. Med. Sci. Sports Exerc. 27, 1086–1089. 10.1249/00005768-199507000-00019 7564977

[B58] Stefani R. (2012). Olympic swimming gold: The suit or the swimmer in the suit? Significance 9, 13–17. 10.1111/j.1740-9713.2012.00553.x

[B59] SwimNews (2011). Best Performances Database. Available online at: https://www.swimnews.com (accessed June 10, 2011).

[B60] Takagi H. Sanders R. (2000). Hydrodynamics makes to splash. Physics Word 9, 39–43. 10.1088/2058-7058/13/9/30

[B61] Toussaint H. M. Bruinink L. Coster R. Looze M. Rossem B. V. Van Veenen R.. (1989). Effect of a triathlon wet suit on drag during swimming. Med. Sci. Sports Exerc. 21, 325–328. 10.1249/00005768-198906000-00017 2733583

[B62] Toussaint H. M. Truijens M. Elzinga M. de Ven A. V. de best H. Snabel B.. (2002). Swimming: effect of a fast-skin ‘body’ suit on drag during front crawl swimming. Sports Biomech. 1, 1–10. 10.1080/14763140208522783 14658132

[B63] Trappe T. A. Starling R. D. Jozsi A. C. Goodpaster B. H. Trappe S. W. Nomura T.. (1995). Thermal responses to swimming in three water temperatures: Influence of a wet suit. Med. Sci. Sports Exerc. 27, 1014–1021. 10.1249/00005768-199507000-00010 7564968

[B64] Van Geer E. Molenbroek J. Schreven S. de Voogd-Claessen L. Toussaint H. M. (2012). Comparing swimsuits in 3D. Work 41, 4025–4030. 10.3233/WOR-2012-0066-4025. 22317338

[B65] Wada T. Sato T. Ohishi K. Tago T. Izumi T. Matsumoto T. (2010). An analysis of the underwater gliding motion in collegiate competitive swimmers. Biomech. Med. Swim. XI, 185–187. 10.1249/01.MSS.0000385910.34341.6a

[B66] Yermahanova A. Nurmakhambetova D. Bozhig Z. Imanbetov A. (2016). Evaluation of features of development of sports way of swimming of students of various sports specialization. Int. J. Environ. Sci. Educ. 11, 10895–10904.

